# High Neutrophil-to-Lymphocyte and Platelet-to-Lymphocyte Ratios Are Associated with a Higher Risk of Hemodialysis Vascular Access Failure

**DOI:** 10.3390/biomedicines10092218

**Published:** 2022-09-07

**Authors:** Edoardo Pasqui, Gianmarco de Donato, Elisa Lazzeri, Cecilia Molino, Giuseppe Galzerano, Michele Giubbolini, Giancarlo Palasciano

**Affiliations:** Vascular Surgery Unit, Department of Medicine, Surgery and Neuroscience, University of Siena, 53100 Siena, Italy

**Keywords:** end-stage renal disease, hemodialysis, inflammation, biomarkers, arterio-venous fistula

## Abstract

Our aim was to determine the predictive role of the preoperative neutrophil-to-lymphocyte ratio (NLR) and platelet-to-lymphocyte ratio (PLR) in vascular access malfunctioning in patients who had undergone their first native arterio-venous fistula (AVF) for hemodialysis. Methods: This was a single-center retrospective observational study. All patients who underwent the procedure of the creation of a first native AVF for hemodialysis from January 2019 to December 2020 were considered eligible to be part of this study. Reinterventions for AVF malfunctioning were registered and the population was subdivided into two groups with respect to AVF malfunctioning. ROC curves were obtained to find the appropriate cut-off values for the NLR and PLR. A multivariate analysis was used to identify the independent predictors for an AVF malfunction. Kaplan–Meier curves were used to evaluate the AVF patency rates. A total of 178 patients were enrolled in the study, of them 70% (*n* = 121) were male. The mean age was 67.5 ± 12 years. Reinterventions for AVF malfunctioning were performed on 102 patients (57.3%). An NLR > 4.21 and a PLR > 208.8 was selected as the cut-off for AVF malfunctioning. The study population was divided into two groups depending on the NLR and PLR values of the individual. For the NLR < 4.21 group, the AVF patency rates were 90.7%, 85.3%, and 84% at the 3-, 6-, and 12-month follow-up, respectively, and 77.5%, 65.8%, and 39.3% at 3, 6, and 12 months for the NLR > 4.21 group, respectively (*p* < 0.0001). For the PLR < 208.8 group, the patency rates were 85.6%, 76.7%, and 67.7% at the 3-, 6-, and 12-month follow-up. For the PLR > 208.28 group, the patency rates were 80.8%, 71.2%, and 50.7% for the 3-, 6-, and 12-month follow-up, respectively (*p* = 0.014). The multivariate analysis highlighted that diabetes mellitus, the neutrophil count, the lymphocyte count, and the NLR were independent risk factors for an AVF failure. In our experience, the NLR and PLR are useful markers for the stratification of vascular access failure in hemodialysis patients. The inexpensive nature and ready availability of the values of these biomarkers are two points of strength for everyday clinical practice.

## 1. Introduction

The number of patients with end-stage chronic kidney disease is growing day by day and, consecutively, so is the need for hemodialysis. The Kidney Disease Outcomes Quality Initiative (K/DOQI) [1] and the European Society of Vascular and Endovascular Surgery Guidelines [2] recommend an autologous arterio-venous fistula (AVF) as the primary and preferred vascular access in performing hemodialysis thanks to a higher quality of life and fewer complications for patients in comparison with the alternatives available. Nevertheless, AVFs are prone to failure due to stenosis and thrombosis, leading to reinterventions, the creation of a new vascular access, or the positioning of dedicated catheters. Numerous elements concur with an AVF failure. Inflammation is one of them, determining the neointimal hyperplasia, stenosis, and procoagulative state [3]. Exploring the role of inflammatory markers such as the neutrophil-to-lymphocyte ratio (NLR) and platelet-to-lymphocyte ratio (PLR) could reveal the subclinical systemic inflammatory status that could lead to a premature AVF failure. The NLR and PLR have already been linked to several cardiovascular pathologies [4,5,6] and surgical outcomes [7,8,9]. In this light, our aim was to determine the predictive role of the preoperative NLR and PLR in vascular access malfunctioning in patients who had undergone their first native AVF for hemodialysis.

## 2. Materials and Methods

All patients who underwent the procedure of the creation of a first native AVF for hemodialysis from January 2019 to December 2020 were considered eligible to be part of this study.

Every hospitalization was retrospectively analyzed in terms of the anamnestic features, medications, preoperative blood samples (white blood count, neutrophil and lymphocyte subpopulation, and platelets), and types of procedures performed.

In detail, the biochemical inflammatory markers were analyzed, calculating the NLR and PLR. The NLR and PLR were obtained by dividing the absolute neutrophil and platelet counts by the lymphocyte count. The SII was calculated using the following formula: (neutrophil count x platelet count)/lymphocyte count. 

The inclusion criteria consisted of patients who underwent an autogenous radio-cephalic AVF and an autogenous brachiocephalic AVF creation and who had available preoperative complete blood samples that had been measured in the institutional laboratory. All patients had to begin hemodialysis at the moment of the AVF creation and did not experience central venous catheterization. The exclusion criteria consisted of patients who had any condition that could have affected the white blood cell count and differential counts such as an immediate past, a current history, or signs or symptoms of an infection; hematologic diseases; and recent (14 days or less) steroid intake. In addition, patients with AVFs opened with a synthetic graft were not included in the study.

Patients whose dialysis started later than 3 months after the AVF creation as well as those who were lost to follow-up during the first 3 months after surgery or had incomplete data were also excluded.

Follow-up evaluations were performed at 2 weeks after the AVF surgery and then every month for an additional 3–6 months to monitor the AVF success or failure as well as complications. The follow-up examinations were performed by experienced vascular surgeons using a duplex ultrasound. Malfunctions that occurred after AVF maturation were highlighted by the nephrologists during the hemodialysis sessions. In the case of complications, patients were referred for a surgical re-evaluation. If an AVF failed to function or any complications occurred, an additional intervention was performed.

The ethical committee was informed of the no-experimental design of the retrospective investigation and endorsed the study. An informed consent waiver was approved by the ethical committee due to the retrospective design of this study based on patient records. We performed the study in accordance with the Declaration of Helsinki.

### 2.1. Definitions and Outcomes

The primary access functional patency is related to the functionality of the access and its continuous use for successful hemodialysis during its indicated patency. An access is said to be “functional” when it is able to deliver a flow rate of 350 to 400 mL/min without access to recirculation to maintain a treatment time less than 4 h. Thus, primary patency was defined as the interval from the time of the access placement to the time of any intervention designed to maintain or re-establish patency, access a thrombosis, or the measurement of patency [10].

Reinterventions were defined as any surgical, endovascular, or hybrid manipulation of the access to maintain or re-establish its functionality. 

### 2.2. Statistical Analysis

The categorical data were reported as numbers and percentages. The means (±standard deviation) were used to analyze the continuous variables. A Student’s *t*-test was used to compare the normally distributed variables. Mann–Whitney U tests were used to compare the non-normally distributed variables. The categorical variables were compared using a Fisher’s exact test. The statistical significance was considered to be a *p*-value of less than 0.05.

An analysis of the NLR and PLR for vascular access failure was conducted.

The NLR and PLR were studied by receiver operating characteristic (ROC) curves with vascular access failure. Optimal cut-offs were chosen based on the specificity and sensitivity.

Vascular access patency was estimated using the Kaplan–Meier method and the NLR and PLR cut-offs obtained by the ROC curves. The log-rank test was used to compare all life table curves performed in the study.

The datasets were analyzed using univariate methods with the aim of determining the risk factors correlated to the vascular access loss of patency.

A Cox proportional analysis was used to determine the independent predictors for vascular access failure; a probability of 0.10 was used to enter the variables into the Cox model in a forward-stepwise manner. A probability of 0.15 was used to remove the variables from the model. Independent predictor variables that contributed to the final multivariate model were considered to be significant risk factors for restenosis if they achieved a two-sided *p* < 0.05.

All statistical analyses were performed with GraphPad Prism 9.0 (GraphPad Software Inc., San Diego, CA, USA) and StatPlus Build 7.1.1 (AnalysisSoft Inc., Walnut, CA, USA).

## 3. Results

**Figure 1:**
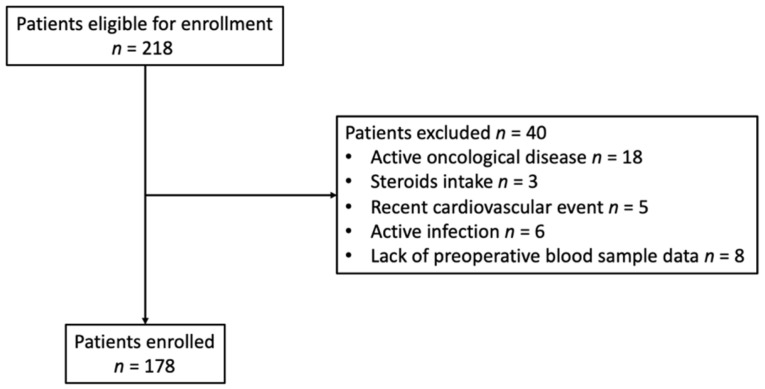
Flow diagram of study population enrollment.

A total of 218 patients were initially enrolled in the study. Of them, 40 were excluded: 18 had an active oncological disease; 3 had assumed steroids; 5 had an acute cardiovascular event that had occurred in the previous month; 6 had an active infection; and the remaining 8 were excluded due to the lack of a preoperative blood sample (Figure 1). 

A total of 178 patients were enrolled in the study, of them 70% (*n* = 121) were male. The mean age was 67.5 ± 12 years. A full list of the baseline preoperative characteristics is provided in Table 1.

The most common causes of end-stage renal disease were hypertension (45.5%), glomerulonephritis (23.6%), and diabetes mellitus (21.3%); in 9.6%, the cause was not defined. 

In 67.4% (*n* = 115), a native distal AVF fistula was created whereas in 32.6% (*n* = 63), a proximal native AVF fistula was created. 

Reinterventions were performed on 102 patients (57.3%). In 54 patients (30.3%), multiple reinterventions were registered. The causes of the first reinterventions were: AVF thrombosis in 46 cases (45.1%); AVF stenosis in 43 cases (42.2%); and central vein stenosis/occlusion in 13 cases (12.7%). In 70.6% (72/102), a surgical/endovascular AVF revision was performed; in the residual 30 cases (29.4%), a new AVF was created. 

### 3.1. Inflammatory Marker Results

The baseline laboratory data are reported in Table 1.

**Figure 2:**
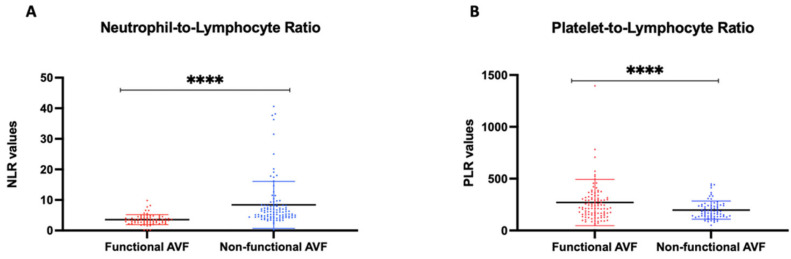
Biochemical marker differences between AVF functioning and AVF malfunctioning groups. (**A**) Neutrophil-to-Lymphocyte Ratio mean value and Standard deviation between Functional and Non-Functional Arterovenous fistula; (**B**) Platelet-to-Lymphocyte Ratio mean value and standard deviation between Functional and Non-Functional Arterovenous fistula. Asterisks indicated the highly significant differences between values.

The mean NLR was 6.32 ± 3.12 whereas the mean PLR was 238.92 ± 105.23. The mean values of both the NLR and PLR were statistically different between the groups of functioning and non-functioning AVFs: NLR 3.55 ± 1.62 vs. 8.27 ± 6.7 (*p*-value = 0.0001) and PLR 194 ± 89.7 vs. 266 ± 170 (*p*-value = 0.001), respectively. (Figure 2)

The single cut-offs for the NLR and PLR were calculated and ROC curves were obtained to analyze the effect of the NLR and PLR with vascular access malfunctioning. 

**Figure 3:**
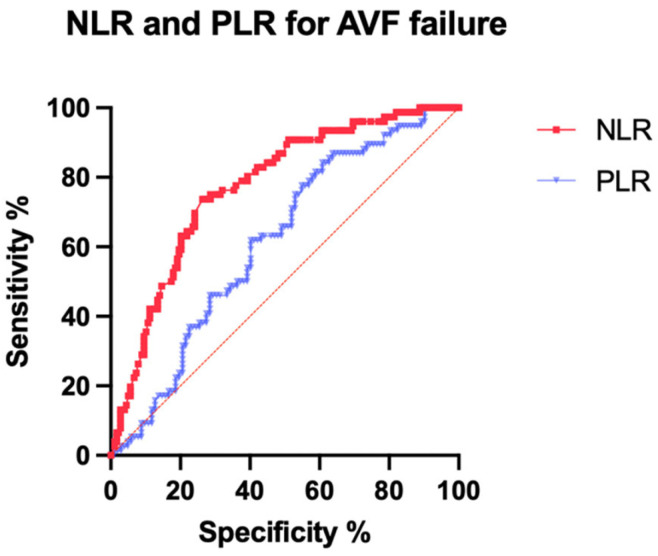
Receiver operator characteristic (ROC) curves. Red curve: NLR for arterio-venous failure; blue curve: PLR for arterio-venous failure.

The ROC curves identified the following values (Figure 2): an NLR > 4.21 was selected as the cut-off for AVF malfunctioning (sensitivity of 75% (95% CI 64.22–83.37%) and specificity of 69.66% (95% CI 62.55–75.94%)) with an area under the curve (AUC) of 0.7733 (95% CI 0.7128–0.8339; *p* < 0.0001); a PLR > 208.8 was selected as the cut-off for AVF malfunctioning (sensitivity of 61.84% (95% CI 50.6–71.94%) and specificity of 56.86% (95% CI 47.517–66.05%)) with an AUC of 0.6131 (95% CI 0.5308–0.6954; *p* = 0.009). (Figure 3).

**Figure 4:**
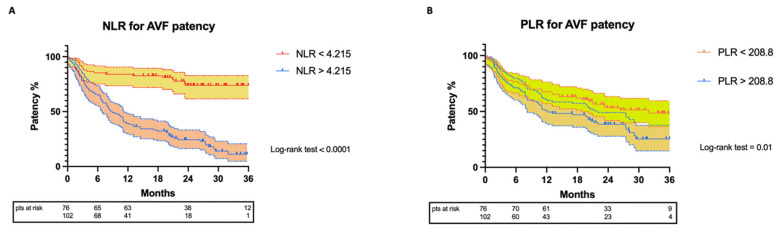
Kaplan–Meier curves representing: (**A**) arterio-venous fistula patency based on NLR cut-off value; (**B**) arterio-venous fistula patency based on PLR cut-off value. Color-filled areas represent the standard error.

The study population was divided into two groups, depending on the NLR and PLR values of the individual. Kaplan–Meier survival curves for AVF patency were obtained. (Figure 4) 

For the NLR < 4.21 group, the patency rates were 98.6%, 90.7%, 85.3%, and 84% at the 1-, 3-, 6-, and 12-month follow-up, respectively. For a longer follow-up, the patency rate was 73.9% at 24 and 36 months. For the NLR > 4.21 group, the patency rates were 94.2%, 77.5%, 65.8%, and 39.3% at the 1-, 3-, 6-, and 12-month follow-up, respectively. The patency rates were 24.4% and 11.2% at the 24- and 36-month follow-up, respectively. The log-rank test revealed that the two curves were significantly statistically different (*p* < 0.0001).

The same analysis was repeated for the PLR. 

For the PLR < 208.8 group, the patency rates were 94.5%, 85.6%, 76.7%, and 67.7% at the 1-, 3-, 6-, and 12-month follow-up, respectively. For a longer follow-up, the patency rates were 53.3% and 48.5% at 24 and 36 months, respectively.

For the PLR > 208.28 group, the patency rates were 96.4%, 80.8%, 71.2%, and 50.7% at the 1-, 3-, 6-, and 12-month follow-up, respectively. The patency rates were 38.5% and 25.5% at the 24- and 36-month follow-up, respectively. The log-rank test revealed that the two curves were significantly statistically different (*p* = 0.014).

### 3.2. Multivariate Analysis

The variables identified as significant from the univariate analyses were entered into the Cox regression analysis (Table 2).

Diabetes mellitus (hazard ratio 1.41 (1.04–2.15)), the neutrophil count (hazard ratio 1.93 (1.34–2.81)), the lymphocyte count (hazard ratio 1.59 (1.34–2.17)), and the NLR (hazard ratio 2.53 (1.89–3.11)) were independent risk factors for an AVF failure.

## 4. Discussion

In this study, we have shown that inflammatory markers such as the NLR and PLR can be informative and helpful tools in relation to AVF malfunctioning. 

We have demonstrated that a higher preoperative NLR and PLR are associated with reduced AVF patency. Although the NLR was found to be an independent risk factor for an AVF failure, the PLR was not. 

End-stage chronic kidney disease is increasing in terms of incidence and prevalence worldwide. The creation and maintenance of a reliable vascular access for hemodialysis is essential, and an autologous AVF is recommended as the first-line choice. Unfortunately, AVFs are prone to several complications due to the continuous traumatism related to the punctures of the hemodialysis needle as well as to the hemodialysis sessions themselves. AVF stenosis and thrombosis are the most important causes of failure, leading to reinterventions, the creation of a new AVF, or central vein catheter positioning.

The real mechanism of these events is not clearly defined, but inflammation is advocated as one of the most important and influential factors that can lead to vascular access malfunctioning. 

From this perspective, inflammation has recently been linked to neointimal hyperplasia, which is one of the most common causes for AVF stenosis and thrombosis. 

In several chronic debilitating disorders, such as chronic kidney disease, inflammation becomes maladaptive, uncontrolled, and persistent. Systemic persistent inflammation has, for almost 20 years, been recognized as a major contributor to the uremic phenotype and a predictor of cardiovascular and total mortality. Moreover, inflammation is likely to be the consequence of a multifactorial etiology and interacts with several factors that emerge when uremic toxins accumulate [11,12].

The identification of reliable markers for the systemic inflammation status could be useful in stratifying the risk of an AVF failure or malfunction. Several markers have been studied in order to understand what type of patients are more prone to develop AVF stenosis and subsequent failures. 

Most of these markers are expensive and are not usually used in daily clinical practice. Specifically, interleukin-6, interleukin-1L-β, and C-reactive protein have been studied in a few exploratory experiences with data that confirm a potential link between inflammation and the vascular access patency [13,14,15,16]. In this light, the validation of inexpensive and readily available biomarkers could enlighten interesting clinical scenarios. The NLR is becoming an important tool for clinical practice as a surrogate of the systemic inflammation index. It has already been linked with several clinical scenarios such as cardiovascular [17,18,19], oncological [20,21], and many other medical and surgical fields [22,23]. 

In the literature, there are a few preliminary experiences in which the NLR has been related to vascular access maturation and complications, sometimes with conflicting results. 

Bashar et al., proposed that patients with a higher level of NLR were more likely to have a matured AVF than patients with a lower NLR level [24]; however, a more recent experience revealed that a preoperative and postoperative NLR value > 2.5 and 2.7, respectively, were associated with an AVF failure [25]. The latter results were confirmed by another recent experience in which the NLR was advocated as a reliable biomarker for vascular access complications. The NLR and red blood cell distribution width were found to be strong independent predictors of an AVF failure; in detail, an NLR value > 2.65 had a specificity of 80% and a sensitivity of 98% in predicting vascular access failures in a cohort of 150 patients [26]. An NLR > 2.7 was also associated with late AVF stenosis [27]. In addition, Çildağ et al., found that the NLR could influence the primary patency rate of an AVF after a balloon angioplasty in the treatment of restenosis, especially when using conventional ballooning [28]. These emerging data are in line with our results, even if we found a higher optimal cut-off value for the NLR than the previous experiences. At one year after the creation of a native AVF, patients with a higher level of inflammation (NLR > 4.21) have more than a three-fold risk of having a malfunctioning vascular access. In the nephrological setting, the PLR has also been explored in various medical fields as an additional inflammatory marker. In a recent experience, the PLR emerged as a factor associated with a high risk of mortality in both peritoneal and hemodialysis patients [29,30].

Very few experiences have investigated the influence of the PLR on vascular access maturation and AVF longevity. In this light, high levels of the PLR may be a supportive finding of AVF stenosis and thrombosis, and may be taken into consideration during the follow-up of hemodialysis-dependent patients [31].

The scientific interest in valid inflammatory biomarkers in predicting the probability of vascular access failure is growing, especially because, in hemodialysis patients, the functionality of an AVF is essential. It is not easy to translate this scientific experience into daily clinical practice in favor of the perspective of the patient. High-AVF-failure-risk patients could be included in a stricter program and follow-up protocol in which the health of the vascular access is monitored with greater attention. Patients could be educated and checked in order to reduce the factors that could increase the level of systemic inflammation such as smoking and alcohol consumption. The punctures of the AVF could be ultrasound-guided in order to reduce unintentional and additional AVF traumas. Finally, the NLR and PLR could be included in a more comprehensive assessment score to increase their potential in predicting an AVF failure or complications, anticipating pre-emptive actions.

### Limitations

Our study has a few limitations. First, the retrospective and observational nature of the study that could preclude the opportunity to derive direct cause-and-effect risk associations; second, the limited numerosity of the study population; third, the impossibility to take into account other inflammatory markers (such as C-reactive protein) that are not usually evaluated before the execution of this type of surgical procedure; and lastly, the predictors in this analysis were assessed only in a single time-point.

## 5. Conclusions

In our experience, the NLR and PLR are useful markers for the stratification of vascular access failure in hemodialysis patients. The inexpensive nature and ready availability of these biomarker values are two points of strengths for their use in everyday clinical practice. Further studies are needed to validate these findings and to define the most accurate cut-offs for the risk stratification for patients and for the definition of customizing surveillance protocols.

## Figures and Tables

**Table 1:** Baseline characteristics of the population study.

Variables	Total Population	Functional AVF Group (*n* = 76)	Non-Functional AVF Group (*n* = 102)	*p*-Value
	(*n* = 178)			
**Age (Mean ± SD)**	67.5 ± 12	68.5 ± 13.2	66.1 ± 12.5	0.2
**Male (n, %)**	120 (67.4%)	52 (68.4%)	68 (66.7%)	0.8
**Diabetes Mellitus (n, %)**	36 (20.2%)	10 (13.2%)	26 (25.5%)	**0.05**
**Hypertension (n, %)**	150 (84.3%)	67 (88.2%)	83 (81.4%)	0.3
**Coronary Artery Disease (n, %)**	63 (35.4%)	21 (27.6%)	41 (40.2%)	0.1
**Atrial Fibrillation**				
**Peripheral Artery Disease (n, %)**	16 (9%)	8 (10.5%)	8 (7.8%)	0.6
**COPD**	13 (7.3%)	5 (6.6%)	8 (7.8%)	0.9
**History of Smoking**	26 (14.6%)	8 (10.5%)	18 (17.6%)	0.2
**Cerebrovascular Disease (n, %)**	13 (7.3%)	4 (5.2%)	9 (8.8%)	0.4
**Dyslipidemia (n, %)**	89 (50%)	34 (44.7%)	55 (53.4%)	0.3
**Type of AFV Configuration (n, %)**				
**Distal**	120 (67.4%)	52 (68.4%)	68 (66.7%)	0.8
**Proximal**	58 (32.6%)	24 (31.6%)	34 (33.3%)	0.8
**Type of Puncture For Hemodialysis**				
**Single Needle**	15 (8.4%)	7 (9.2%)	8 (7.8%)	0.8
**Double Needle**	163 (91.6%)	69 (90.8%)	94 (92.2%)	0.8
**Medications**				
**Single Antiplatelet Therapy**	102 (57.3%)	41 (53.9%)	61 (59.8%)	0.4
**Dual Antiplatelet Therapy**	23 (12.9%)	10 (13.2%)	13 (12.7%)	0.9
**Anticoagulants**	15 (8.4%)	6 (7.9%)	9 (8.8%)	0.9
**Statins**	75 (42.1%)	28 (36.8%)	47 (46%)	0.2
**Laboratory Preoperative Variables (Mean ± SD)**				
**Neutrophil (1000/mL)**	5.8 ± 3.11	4.39 ± 2.16	6.85 ± 3.30	**0.0001**
**Lymphocyte (1000/mL)**	1.18 ± 0.51	1.33 ± 0.52	1.06 ± 0.47	**0.0004**
**Platelet (1000/mL)**	229 ± 79.4	236 ± 82.1	225 ± 77.5	0.36
**NLR**	6.32 ± 4.2	3.55 ± 1.62	8.27 ± 6.7	**0.0001**
**PLR**	239 ± 160	194 ± 89.7	266 ± 170	**0.001**
**Creatinine (mg/dL)**	7.6 ± 2.1	7.2 ± 1.8	7.8 ± 2.4	0.07
**Urea (mg/dL)**	256 ± 56	248 ± 42	259 ± 32	**0.03**

Bold *p*-values indicate a statistical significance. COPD: chronic obstructive pulmonary disease; AVF: arterio-venous fistula; NLR: neutrophil-to-lymphocyte ratio; PLR: platelet-to-lymphocyte ratio.

**Table 2:** Stepwise analysis for the predictors of AVF malfunctioning.

Variables for AFV Malfunctioning	Hazard Ratio	CI 95%	*p*-Value
**Age**	0.97	(0.87–1.23)	0.51
**Male**	0.87	(0.78–1.45)	0.63
**Diabetes Mellitus**	1.19	(0.97–1.34)	**0.09**
**Hypertension**	0.9	(0.81–1.32)	0.45
**Coronary Artery Disease**	1.2	(0.98–1.45)	0.67
**Atrial Fibrillation**			
**COPD**	0.94	(0.76–1.29)	0.75
**History of Smoking**	1.13	(0.94–1.76	0.34
**Peripheral Artery Disease**	1.13	(0.67–1.23)	0.73
**Cerebrovascular Disease**	0.89	(0.76–1.34)	0.63
**Dyslipidemia**	0.9	(0.87–1.23)	0.59
**Distal AFV Configuration**	0.85	(0.72–1.17)	0.61
**Single Needle Puncture For Hemodialysis**	0.76	(0.71–1.21)	0.65
**Medications**			
**Single Antiplatelet Therapy**	0.81	(0.65–1.31)	0.71
**Dual Antiplatelet Therapy**	1.03	(0.78–1.25)	0.45
**Anticoagulants**	0.92	(0.76–1.31)	0.67
**Statins**	0.78	(0.56–1.21)	0.54
**Laboratory Preoperative Variables**			
**Neutrophil (1000/mL)**	2.12	(1.45–2.56)	**0.01**
**Lymphocyte (1000/mL)**	1.84	(1.32–2.23)	**0.04**
**Platelet (1000/mL)**	1.61	(1.18–2.72)	**0.08**
**NLR**	2.53	(1.85–2.96)	**0.02**
**PLR**	2.37	(1.64–2.76)	**0.04**
**Creatinine (mg/dL)**	1.21	(1.02–1.45)	**0.05**
**Urea (mg/dL)**	0.98	(0.74–1.45)	**0.43**
**Stepwise (Multivariate) Analysis**			
**Diabetes Mellitus**	1.41	(1.04–2.15)	**0.04**
**History of Smoking**			
**Neutrophil (1000/mL)**	1.93	(1.34–2.81)	**0.04**
**Lymphocyte (1000/mL)**	1.59	(1.34–2.17)	**0.05**
**Platelet (1000/mL)**	1.78	(1.26–2.76)	0.07
**NLR**	2.53	(1.89–3.11)	**0.01**
**PLR**	1.48	(0.98–2.36)	0.06
**Creatinine**	1.21	(0.87–1.45)	0.63

Bold *p*-values indicate a statistical significance. AVF: arterio-venous fistula; COPD: chronic obstructive pulmonary disease; NLR: neutrophil-to-lymphocyte ratio; PLR: platelet-to-lymphocyte ratio.

## Data Availability

The data presented in this study are available on request from the corresponding author. The data are not publicly available due to privacy issue.
